# Causes of Visual Loss in Uveitis: A Retrospective Study at a Tertiary Referral Centre

**DOI:** 10.7759/cureus.102183

**Published:** 2026-01-23

**Authors:** Mohammed Al-Roubaie, Nicholas Beare

**Affiliations:** 1 Ophthalmology, Northwick Park Hospital, London, GBR; 2 Ophthalmology, Liverpool University Hospitals NHS Foundation Trust, Liverpool, GBR

**Keywords:** blindness, cataract, cystoid macular oedema, epidemiology, uveitis, visual loss

## Abstract

Purpose: The purpose of the study is to determine the causes of visual loss in patients with uveitis attending a tertiary referral clinic and to assess the relative contribution of uveitis-related inflammation versus its complications, stratified by uveitis subtype.

Methods: This retrospective, cross-sectional study reviewed the medical records of 442 patients attending the uveitis service at St Paul’s Eye Unit, Royal Liverpool University Hospital. Demographic data, uveitis classification, laterality, systemic treatment, and visual outcomes were extracted from MediSIGHT, an electronic medical records system. Visual impairment was classified using the most recent recorded visual acuity, and causes of visual loss were analysed descriptively.

Results: Among 442 patients (mean age 52 years; 51.6% female), 148 (33.5%) had visual loss in at least one eye, including 64 (14.5%) with moderate visual impairment, 13 (2.9%) who were sight-impaired, and 71 (16.1%) who were severely sight-impaired. Cystoid macular oedema (33.1%) and cataract or aphakia (24.3%) were the most common causes, together accounting for 57.4% of visual loss. Visual loss occurred most frequently in posterior uveitis (53.1%), compared with anterior uveitis (19.6%; *p* < 0.001). Bilateral inflammation was associated with higher rates of visual loss than unilateral disease (41.3% vs 26.9%; *p* = 0.002).

Conclusion: Visual loss remains a common outcome in patients with uveitis, most frequently arising from disease-related complications such as cystoid macular oedema and cataract. Posterior uveitis and panuveitis are associated with the highest risk of impairment. These findings highlight the ongoing burden of uveitis-related visual morbidity within tertiary care settings.

## Introduction

Uveitis is a term used to describe a group of relatively uncommon conditions that are characterised by inflammation of the uvea, the vascular middle layer of the eye that lies between the retina and the sclera [1]. These conditions are not limited to the uveal tract and may affect the retina, the retinal blood vessels ("retinal vasculitis"), and other tissues [2]. Uveitis can occur at any age but often affects patients in the working age group [2,3]. There are four types of uveitis: anterior (maximal inflammation of the anterior chamber and iris), intermediate (maximal inflammation of the ciliary body and pars plana, manifests as vitritis in the posterior chamber of the eye), posterior (inflammation of the choroid and retina) and panuveitis (simultaneous inflammation of the anterior and posterior chamber), with intermediate uveitis being the least common. Uveitis can be non-infectious or infectious. Non-infectious uveitis can be associated with systemic inflammatory conditions, such as sarcoidosis, seronegative spondyloarthropathies and vasculitides, or may be purely ocular. Infectious causes include syphilis, tuberculosis and herpes anterior uveitis or viral retinitis [4].

The average annual incidence of uveitis has been reported as approximately 14-17 per 100,000, rising to a peak in the 20-50 age group before declining, with an estimated prevalence of 58.0 to 114.5 per 10,000 persons [5]. Despite this, uveitis accounts for an estimated 10% to 15% of all cases of blindness in the developed world. Additionally, uveitis is responsible for up to 25% of instances of legal blindness in developing countries [6]. It is uncommon in children under 10 years of age and in adults over 70 years of age [7]. The majority of epidemiological research on uveitis has been conducted at tertiary referral centres and is therefore biased toward posterior and intermediate uveitis, which are typically more severe but less common than anterior uveitis. One study conducted in a primary care setting demonstrated that anterior uveitis accounted for up to 90% of cases [8].

The aims of this retrospective, non-interventional study were to determine the frequency and causes of visual loss in patients with uveitis attending a tertiary referral clinic, to characterise the clinical mechanisms associated with visual loss (including inflammatory activity and uveitis-related structural complications), and to examine how the risk and causes of visual loss vary according to the anatomical subtype and aetiology of uveitis. The burden of visual loss due to uveitis is likely underestimated because of poor representation in the certification of vision impairment process and epidemiological surveys. This is compounded by complications of uveitis (e.g. secondary glaucoma) being categorised as the primary cause of visual loss, highlighting the importance of understanding patterns of causes of visual loss in uveitis. 

Uveitis is associated with a range of structural complications that frequently lead to vision impairment. Common complications include cataract formation, glaucoma, epiretinal membrane formation, neovascularisation (both retinal and choroidal), and anterior synechiae [9]. With a majority of patients in the working age group, the potential social and economic burden is substantial. For a disease with such far-reaching consequences, relatively few data exist about the morbidity caused by uveitis [3].

Visual morbidity does not typically develop as a result of a single incident of uveitis; rather, repeated bouts of inflammation produce cumulative damage. However, certain individuals with severe illness may develop refractory cystoid macular oedema early on, while those with Behçet's disease may experience catastrophic visual loss within days from occlusive retinal vasculitis despite rigorous immunosuppression [10]. 

## Materials and methods

Study design and setting

This was a retrospective, cross-sectional observational study conducted at St Paul’s Eye Unit, a tertiary referral uveitis service at the Royal Liverpool University Hospital, United Kingdom. The service receives referrals from a wide geographical catchment area including North Wales, Lancashire, South Cumbria, Cheshire, and Merseyside. The study utilised routinely collected clinical data and was registered locally as a service evaluation. In accordance with institutional policy, formal ethical approval and individual patient consent were not required.

Study population

Medical records of 450 consecutive patients attending the uveitis service were reviewed. Following application of inclusion and exclusion criteria, 442 patients with a confirmed diagnosis of uveitis were included in the final analysis. Patients were excluded if they did not have a confirmed uveitis diagnosis, had no recorded follow-up visits, or had visual loss attributable to causes unrelated to uveitis.

Data collection

Patient data were extracted from MediSIGHT, a proprietary electronic medical record system used for routine clinical documentation. No proprietary algorithms or licensed scoring tools were analysed. Variables collected included patient age, sex, ethnicity, anatomical classification of uveitis, underlying aetiology, laterality of inflammation, systemic and local treatments, and visual acuity measurements. All patients had undergone a comprehensive ophthalmic assessment as part of routine clinical care, including presenting visual acuity measurement with spectacle or contact lens correction where appropriate, slit-lamp biomicroscopy, intraocular pressure measurement, and indirect ophthalmoscopy.

Visual acuity and outcome definitions

Visual acuity was recorded using standard Snellen notation, a universally adopted and freely available clinical measure of visual function [11]. Visual acuity used for analysis was taken from the most recent documented clinic visit. Visual impairment was classified using Snellen thresholds consistent with UK certification criteria for visual impairment [12]. Patients were categorised as having no visual impairment if the worst-seeing-eye visual acuity was 6/12 or better. Moderate visual impairment was defined as visual acuity worse than 6/12 and up to 6/36. Sight impairment was defined as visual acuity worse than 6/36 and up to 3/60, while severe sight impairment was defined as visual acuity worse than 3/60.

Functional visual status was additionally assessed based on binocular vision where relevant. Causes of visual loss were classified as either direct inflammatory involvement, such as chorioretinitis, or uveitis-related complications.

Anatomical classification

Anatomical classification of uveitis was defined according to the Standardisation of Uveitis Nomenclature (SUN) Working Group criteria [13].

Statistical analysis

Data were analysed descriptively to characterise the study population and causes of visual loss. Categorical variables were summarised as frequencies and percentages. Comparisons between anatomical subtypes were performed using the chi-squared test. Statistical significance was defined as a p-value less than 0.05.

## Results

A total of 442 patients with uveitis were included in the study after application of inclusion and exclusion criteria, with eight patients excluded due to the absence of a confirmed uveitis diagnosis. The mean age was 52 years (range 20-96 years), and there were 228 female patients (51.6%) and 214 male patients (48.4%). Ethnicity was recorded for 304 patients (68.8%), of whom 276 (90.8%) were of White ethnicity. Baseline demographic and clinical characteristics of the cohort are summarised in Table 1.

**Table 1:** Baseline demographic and clinical characteristics of the study cohort (N = 442) Data are presented as number (%) unless otherwise stated. Percentages for ethnicity are calculated among patients with ethnicity recorded (n = 304). Anatomical classification was defined according to the Standardisation of Uveitis Nomenclature (SUN) criteria [13].

Characteristic	n (%)
Age (years)	
Mean (range)	52 (20–96)
Sex	
Female	228 (51.6)
Male	214 (48.4)
Ethnicity	
Recorded	304 (68.8)
Not recorded	138 (31.2)
Ethnicity (of those recorded, n = 304)	
White	276 (90.8)
Asian	6 (2.0)
Black	15 (4.9)
Other	7 (2.3)
Anatomical classification (SUN)	
Anterior uveitis	163 (36.9)
Intermediate uveitis	120 (27.1)
Posterior uveitis	96 (21.7)
Panuveitis	18 (4.1)
Not stated	45 (10.2)

Within this cohort of 442 patients, when vision was classified according to the worst-seeing eye, 294 patients (66.5%) had no visual impairment (6/12 or better). The remaining 148 patients (33.5%) demonstrated visual impairment in at least one eye, including 64 (14.5%) with moderate visual impairment (6/18-6/36), 13 (2.9%) who were sight-impaired (6/60-3/60), and 71 (16.1%) who were severely sight-impaired (worse than 3/60).

When functional vision was assessed based on both eyes, 387 patients (87.6%) retained no visual impairment. Functional visual impairment was present in 55 patients (12.4%), comprising 40 (9.0%) with moderate visual impairment, nine (2.0%) who were sight-impaired, and 6 (1.4%) who were severely sight-impaired. These distributions are summarised in Table 2.

**Table 2:** Visual impairment categories by worst-eye and functional vision Visual impairment categories are based on worst-eye Snellen visual acuity and UK certification thresholds for visual impairment [11,12]. Percentages are calculated per patient. VI: visual impairment; MVI: moderate visual impairment; SI: sight-impaired; SSI: severely sight-impaired.

Vision classification	Worst eye (%)	Both eyes “functional vision” (%)
No VI (6/12 or better)	294 (66.5)	387 (87.6)
MVI (6/18 – 6/36)	64 (14.5)	40 (9)
SI (6/60 – 3/60)	13 (2.9)	9 (2)
SSI (< 3/60)	71 (16.1)	6 (1.4)

Amongst the 148 patients with visual loss in at least one eye, the main causes were cystoid macular oedema (n = 49, 33.1%), cataract/aphakia (n = 36, 24.3%) and choroidal neovascularisation (n = 14, 9.5%). Other causes included epiretinal membrane, secondary glaucoma, retinal detachment (tractional, exudative and rhegmatogenous), vitreous opacity or haemorrhage, optic neuropathy, vessel occlusion and band keratopathy. The causes of visual loss are detailed in Table 3.

**Table 3:** Causes of visual loss by worst-eye visual impairment category. Visual impairment categories are based on worst-eye Snellen visual acuity and UK certification thresholds for visual impairment [11,12]. Visual loss refers to patients with moderate visual impairment or worse (n = 148). The No VI column lists ocular complications present in patients whose worst-eye visual acuity remained 6/12 or better at the most recent assessment. VI: visual impairment; MVI: moderate visual impairment; SI: sight-impaired; SSI: severely sight-impaired; RD: retinal detachment.

Cause of visual loss	No VI (6/12 or better)	MVI (6/18 – 6/36)	SI (6/60 – 3/60)	SSI (< 3/60)	Visual loss (n=148), n (%)	Total cohort (N = 442), n (%)
Cystoid macular oedema	66	29	4	16	49 (33.1)	115 (26.0)
Cataract/aphakia	36	16	3	17	36 (24.3)	72 (16.3)
Chorioretinitis	31	1	0	7	8 (5.4)	39 (8.9)
Epiretinal membrane	23	6	0	1	7 (4.7)	30 (6.8)
Secondary glaucoma	17	4	1	4	9 (6.1)	26 (5.9)
Choroidal neovascularisation	8	1	2	11	14 (9.5)	22 (5.0)
Vitreous opacity or haemorrhage	17	3	0	1	4 (2.7)	21 (4.8)
Tractional or exudative RD	3	1	2	4	7 (4.7)	10 (2.3)
Optic neuropathy	1	1	1	3	5 (3.4)	6 (1.4)
Vessel occlusion	3	1	0	2	3 (2.0)	6 (1.4)
Other	3	0	0	1	1 (0.7)	4 (0.9)
Band keratopathy	0	0	0	1	1 (0.7)	1 (0.9)
Ischaemic retina	0	0	0	1	1 (0.7)	1 (0.9)
Rhegmatogenous RD	0	0	0	1	1 (0.7)	1 (0.9)
No identifiable cause	85	2	0	1	3 (2.0)	88 (19.9)

The underlying causes of uveitis were also examined. Idiopathic uveitis, HLA-B27-associated disease, and sarcoidosis were the most common diagnoses across all vision groups. A comprehensive summary of uveitis aetiology is presented in Table 4.

**Table 4:** Causes of uveitis stratified by worst-eye visual impairment category. Visual impairment categories are based on worst-eye Snellen visual acuity and UK certification thresholds for visual impairment [11,12]. Percentages represent proportions of the total study cohort (N = 442). Diagnoses with low frequency are shown to provide a comprehensive overview of uveitis aetiology within this tertiary cohort. VI: visual impairment; MVI: moderate visual impairment; SI: sight-impaired; SSI: severely sight-impaired; POHS: presumed ocular histoplasmosis syndrome; PIC: punctate inner choroidopathy; JIA: juvenile idiopathic arthritis; IBD: inflammatory bowel disease; TB: tuberculosis; VKH: Vogt–Koyanagi–Harada; CMV: cytomegalovirus; HSV: herpes simplex virus; VZV: varicella zoster virus; MEWDS: multiple evanescent white dot syndrome; APMPPE: acute posterior multifocal placoid pigment epitheliopathy; AZOOR: acute zonal occult outer retinopathy; IRVAN: idiopathic retinal vasculitis, aneurysms, and neuroretinitis.

Diagnosis	No VI (6/12 or better)	MVI (6/18 – 6/36)	SI (6/60 – 3/60)	SSI (< 3/60)	Total (N = 442), n	Percentage (%)
Idiopathic	114	26	5	14	159	36.0
Sarcoid	22	9	0	9	40	9.1
HLA B27 alone	28	4	0	1	33	7.5
AS	14	0	0	1	15	3.4
Toxoplasmosis	13	2	0	0	15	3.4
Multi-focal choroiditis	11	1	0	2	14	3.2
MS	9	1	0	3	13	2.9
Fuch’s HI	9	4	0	0	13	2.9
POHS/PIC	9	1	1	2	13	2.9
JIA	7	0	0	4	11	2.5
Behçet’s	4	0	1	4	9	2.0
Birdshot chorioretinopathy	5	2	0	2	9	2.0
Serpingous choroiditis	1	0	2	5	8	1.8
IBD	6	1	0	1	8	1.8
TB uveitis	3	1	1	3	8	1.8
Other infectious	4	2	1	0	9	1.6
Syphilis	5	1	0	0	6	1.4
VKH	4	0	0	2	6	1.4
Sympathetic	0	0	0	6	6	1.4
CMV viral retinitis	1	0	2	2	5	1.1
Psoriasis	2	2	0	0	4	0.9
Other non-infectious	3	0	0	1	4	0.9
HSV viral retinitis	1	0	0	2	3	0.7
Endogenous endophthalmitis	1	0	0	2	3	0.7
GWP (Wegener’s)	3	0	0	0	3	0.7
Neuroretinitis	2	0	0	1	3	0.7
VZV viral retinitis	1	0	0	1	2	0.5
MEWDS	2	0	0	0	2	0.5
Autoimmune retinopathy	0	1	0	1	2	0.5
APMPPE	2	0	0	0	2	0.5
Post-infectious uveitis	0	1	0	0	1	0.2
Lyme disease	0	1	0	0	1	0.2
Pars planitis	1	0	0	0	1	0.2
AZOOR	0	0	0	1	1	0.2
Cancer associated retinopathy	1	0	0	0	1	0.2
IRVAN syndrome	0	0	0	1	1	0.2
Eales Disease	0	0	0	1	1	0.2
Other systemic	0	1	0	0	1	0.2
Reactive arthritis	1	0	0	0	1	0.2

Chronic unilateral intraocular inflammation was present in 212 patients (48%), of whom 57 (26.9%) experienced visual loss. Among these, 29 (50.9%) had moderate visual impairment, 13 (22.8%) were sight-impaired, and 24 (42.1%) were severely sight-impaired. Bilateral inflammation was present in 230 patients (52%), with visual loss occurring in 95 (41.3%). Of these, 35 (36.8%) had moderate visual impairment, 13 (13.7%) were sight-impaired, and 47 (20.4%) were severely sight-impaired.

Visual loss was most frequently observed in posterior uveitis, affecting 51 of 96 patients (53.1%), followed by panuveitis (9/18, 50.0%), intermediate uveitis (41/120, 34.2%), and anterior uveitis (32/163, 24.4%). An additional 15 of 45 patients (33.3%) with unclassified anatomical disease experienced visual loss.

Pairwise comparisons of the proportion of patients with visual loss between anatomical subtypes were performed using Pearson’s chi-squared tests. Visual loss was more common in posterior uveitis than anterior uveitis (53.1% vs. 24.4%, p < 0.001) and intermediate uveitis (53.1% vs. 34.2%, p = 0.005). Panuveitis was also associated with higher rates of visual loss than anterior uveitis (50.0% vs. 24.4%, p = 0.004), with no significant difference between posterior uveitis and panuveitis. Results are summarised in Table 5.

**Table 5:** Visual loss by anatomical classification of uveitis (worst-eye analysis) Visual impairment categories are based on worst-eye Snellen visual acuity [11,12]. Visual loss is defined as moderate visual impairment or worse. Percentages in the final column represent the proportion of patients within each anatomical subgroup who experienced visual loss. Anatomical classification was defined according to the Standardisation of Uveitis Nomenclature (SUN) Working Group criteria [13]. Pairwise comparisons between anatomical subtypes were performed using Pearson’s chi-squared tests. * p = 0.006 compared with anterior uveitis (intermediate vs anterior). † p < 0.001 compared with anterior uveitis (posterior vs anterior). ‡ p = 0.004 compared with anterior uveitis (panuveitis vs anterior). VI: visual impairment; MVI: moderate visual impairment; SI: sight-impaired; SSI: severely sight-impaired.

Anatomical classification	No VI (6/12 or better)	MVI (6/18 – 6/36)	SI (6/60 – 3/60)	SSI (< 3/60)	Total patients, n (%)	Visual loss within subgroup, n (%)
Anterior uveitis	131	20	2	10	163 (36.9)	32 (24.4)
Intermediate uveitis	79	26	2	13	120 (27.1)	41 (34.2)*
Posterior uveitis	45	9	9	33	96 (21.7)	51 (53.1)†
Panuveitis	9	3	0	6	18 (4.1)	9 (50.0)‡
Not stated	30	6	0	9	45 (10.2)	15 (33.3)

Systemic corticosteroid therapy was used in 229 patients (51.8%), with 50 patients (21.9%) receiving doses greater than 10.5 mg of prednisolone. Among patients receiving doses greater than 10.5 mg, 27 (54.0%) experienced visual loss. The distribution of high-dose corticosteroid use across visual impairment categories is shown in Table 6.

**Table 6:** Use of high-dose systemic corticosteroids by visual impairment category. Visual impairment categories are based on worst-eye Snellen visual acuity and UK certification thresholds for visual impairment [11,12]. Percentages represent the proportion of patients within each visual impairment category receiving systemic prednisolone at doses greater than 10.5 mg at the most recent assessment. VI: visual impairment; MVI: moderate visual impairment; SI: sight-impaired; SSI: severely sight-impaired.

Vision classification	Number of patients on > 10.5mg prednisolone (%)
No VI (6/12 or better)	23 (7.8)
MVI (6/18 – 6/36)	4 (6.3)
SI (6/60 – 3/60)	3 (23.1)
SSI (< 3/60)	20 (28.2)

Systemic immunosuppressive therapy other than prednisolone was used in 108 patients (24.4%). Of these, mycophenolate mofetil was the most commonly prescribed agent (50/108, 46.3%), followed by adalimumab (33/108, 30.6%), azathioprine and infliximab (15/108 each, 13.9%), tacrolimus (14/108, 13.0%), ciclosporin (7/108, 6.5%), and sirolimus (3/108, 2.8%). Visual loss occurred in 48 patients receiving systemic therapy (44.4%; p = 0.005). The proportion of patients receiving systemic therapy by vision category is shown in Table 7.

**Table 7:** Use of systemic immunosuppressive therapy (excluding prednisolone) by visual impairment category. Visual impairment categories are based on worst-eye Snellen visual acuity and UK certification thresholds for visual impairment [11,12]. Percentages represent the proportion of patients within each visual impairment category receiving systemic immunosuppressive therapy other than prednisolone at the most recent assessment. VI: visual impairment; MVI: moderate visual impairment; SI: sight-impaired; SSI: severely sight-impaired.

Vision classification	Systemic treatment other than prednisolone (%)
No VI (6/12 or better)	60 (55.6)
MVI (6/18 – 6/36)	15 (23.4)
SI (6/60 – 3/60)	3 (23.1)
SSI (< 3/30)	30 (42.3)

Intravitreal steroid implants (Ozurdex or Iluvien) were administered to 49 patients (11.1%). Among patients with visual loss, implants were used in 15 patients with moderate visual impairment (23.4%), three sight-impaired patients (23.1%), and 12 severely sight-impaired patients (16.9%). These data are summarised in Table 8.

**Table 8:** Use of intravitreal corticosteroid implants by visual impairment category. Visual impairment categories are based on worst-eye Snellen visual acuity and UK certification thresholds for visual impairment [11,12]. Percentages represent the proportion of patients within each visual impairment category who received intravitreal corticosteroid implants (Ozurdex or Iluvien) at any point during follow-up. VI: visual impairment; MVI: moderate visual impairment; SI: sight-impaired; SSI: severely sight-impaired.

Vision classification	Patients receiving Ozurdex or Iluvien, n (%)
No VI (6/12 or better)	19 (6.5)
MVI (6/18 – 6/36)	15 (23.4)
SI (6/60 – 3/60)	3 (23.1)
SSI (< 3/60)	12 (16.9)

## Discussion

The proportion of patients attending our tertiary uveitis clinic with visual loss was high, including 16.1% with severe visual loss. When visual loss was stratified by the underlying mechanism, only a small minority was attributable to direct inflammatory involvement of the retina (chorioretinitis), accounting for 5.4% of cases, while the vast majority resulted from uveitis-related complications. Fewer patients were categorised as sight-impaired than severely sight-impaired, which may reflect the relatively narrow visual acuity range defining sight impairment (6/60-3/60) compared with the open-ended definition of severe sight impairment.

There was a broadly even distribution between men and women, consistent with previous epidemiological studies of uveitis [14,15]. The mean age of the cohort fell within the working-age range. Although studies based on tertiary referral populations have suggested that women may be more susceptible to severe uveitis, this was not observed in the present study, in which a greater proportion of male patients experienced severe sight impairment; however, this difference did not reach statistical significance.

Cystoid macular oedema was the most common cause of visual loss in this cohort, followed by cataract or aphakia. Despite advances in surgical techniques and generally favourable outcomes following cataract surgery in uveitis, cataract remained a frequent cause of visual impairment. This may relate to the increased risks associated with surgery in uveitic eyes or the recommended delay of cataract surgery until inflammation has been quiescent for at least three months [15]. Together, cystoid macular oedema and cataract accounted for over half of all cases of visual loss, consistent with findings from previous studies [16]. Although cystoid macular oedema was classified as a complication in this analysis, it represents a direct consequence of intraocular inflammation, and the distinction between inflammatory activity and secondary complication is not always clear-cut. Choroidal neovascularisation was also an important cause, particularly among patients with severe visual impairment, reflecting its potential for rapid progression to irreversible vision loss.

An unexpected observation was the higher proportion of intermediate uveitis cases compared with posterior uveitis, which contrasts with some published epidemiological series from tertiary referral centres. This likely reflects local referral pathways and disease chronicity rather than true prevalence. Intermediate uveitis is frequently bilateral and chronic, often requiring prolonged subspecialist follow-up for complications such as persistent vitritis and cystoid macular oedema. In contrast, certain forms of posterior uveitis may be acute, self-limiting, or managed through alternative referral pathways, potentially reducing their representation in a general tertiary uveitis clinic cohort. Furthermore, anatomical classification in this study was based on the dominant site of inflammation at the most recent clinic visit and overlap between intermediate and posterior involvement may have resulted in preferential classification as intermediate uveitis in cases with prominent vitritis.

Idiopathic uveitis represented a substantial diagnostic category across all vision groups, which may reflect underlying autoimmune mechanisms or unidentified infectious triggers. Sarcoidosis was also a common cause across multiple categories. Patients without visual impairment were more likely to have idiopathic or HLA-B27-associated disease, which is often characterised by recurrent anterior uveitis and is less commonly associated with permanent visual loss. In contrast, visual loss was more common in intermediate uveitis and highest in posterior uveitis and panuveitis compared with anterior uveitis. This is clinically plausible given the greater likelihood of macular and/or optic nerve involvement and posterior segment complications (e.g., cystoid macular oedema and choroidal neovascularisation), which directly affect central visual acuity.

Patients with more severe visual impairment were more frequently receiving systemic corticosteroid therapy at higher doses, reflecting the greater inflammatory burden in this group. Use of intravitreal steroid implants was more common among patients with moderate visual impairment or sight impairment, particularly in the context of persistent cystoid macular oedema. Patients with severe sight impairment were less frequently treated with intravitreal implants, likely reflecting the presence of irreversible structural macular damage.

Severe sight impairment was associated with a wide range of underlying causes, risk factors, and treatment challenges. Sympathetic ophthalmia was a notable cause in this cohort, with all affected patients experiencing severe visual loss. Recognition of this condition is particularly important, as vision is often already lost in the injured eye, and involvement of the fellow eye can be devastating. Future research may explore strategies to reduce the risk of sympathetic ophthalmia following severe ocular injury.

This study has several limitations. It represents a retrospective, cross-sectional analysis of 442 patients attending a tertiary uveitis service, introducing selection and referral bias toward patients with chronic, complex, or vision-threatening disease, which may limit generalisability to community or primary care populations. The reliance on routinely collected electronic medical record data resulted in incomplete recording of some variables, such as ethnicity, and may have introduced misclassification or missing data bias. Visual acuity was used as the primary outcome measure, which, while standard in epidemiological uveitis research, does not capture other important dimensions of visual function such as contrast sensitivity, visual fields, or patient-reported visual quality. In addition, uveitis represents a heterogeneous group of inflammatory disorders with overlap between anatomical subtypes and aetiological diagnoses; classification in this study was based on the dominant recorded diagnosis and may oversimplify underlying disease complexity. Finally, the cross-sectional design precludes causal inference, and associations between uveitis characteristics, complications, and visual outcomes should not be interpreted as evidence of causation. Despite these limitations, this study provides valuable insight into patterns and causes of visual loss in uveitis within a tertiary care setting.

## Conclusions

Severe visual loss in uveitis is more commonly attributable to disease-related complications rather than direct inflammatory involvement alone. In this cohort, cystoid macular oedema and cataract together accounted for over half of all cases of visual loss. Posterior uveitis and panuveitis were associated with the highest risk of visual impairment. Despite advances in the management of uveitis, a substantial proportion of patients attending tertiary uveitis services continue to experience significant visual loss. Further epidemiological studies that accurately capture uveitis-related visual impairment are required to better define its population-level impact and inform strategies to reduce visual morbidity.
